# Cholesterol promotes EGFR-TKIs resistance in NSCLC by inducing EGFR/Src/Erk/SP1 signaling-mediated ERRα re-expression

**DOI:** 10.1186/s12943-022-01547-3

**Published:** 2022-03-18

**Authors:** Zhenzhen Pan, Kai Wang, Xiniao Wang, Zhirong Jia, Yuqi Yang, Yalei Duan, Lianzhan Huang, Zhuo-Xun Wu, Jian-ye Zhang, Xuansheng Ding

**Affiliations:** 1 School of Basic Medicine and Clinical Pharmacy, China Pharmaceutical University, Nanjing, 211198 Jiangsu China; 2 College of Pharmacy and Health Sciences, St. John’s University, New York, NY 11439 USA; 3 Key Laboratory of Molecular Target & Clinical Pharmacology and the State & NMPA Key Laboratory of Respiratory Disease, School of Pharmaceutical Sciences & The Fifth Affiliated Hospital, Guangzhou Medical University, Guangzhou, 511436 China

**Keywords:** Non-small cell lung cancer, EGFR-TKIs resistance, Cholesterol

## Abstract

**Background:**

The use of epidermal growth factor receptor tyrosine kinase inhibitors (EGFR-TKIs) brings remarkable benefits for the survival of patients with advanced NSCLC harboring EGFR mutations. Unfortunately, acquired resistance seems to be inevitable and limits the application of EGFR-TKIs in clinical practice. This study reported a common molecular mechanism sustaining resistance and potential treatment options to overcome EGFR-TKIs resistance.

**Methods:**

EGFR-TKIs resistant NSCLC cells were established and confirmed by MTT assay. Cholesterol content was detected and the promotional function of cholesterol on NSCLC growth was determined in vivo. Then, we identified ERRα expression as the downstream factor of cholesterol-mediated drug resistance. To dissect the regulatory mechanism, we conducted experiments, including immunofluorescence, co-immunoprecipitation, luciferase reporter assay and chromatin immunoprecipitation assay.

**Results:**

Long-term exposure to EGFR-TKIs generate drug resistance with the characteristic of cholesterol accumulation in lipid rafts, which promotes EGFR and Src to interact and lead EGFR/Src/Erk signaling reactivation-mediated SP1 nuclear translocation and ERRα re-expression. Further investigation identifies ERRα as a target gene of SP1. Functionally, re-expression of ERRα sustains cell proliferation by regulating ROS detoxification process. Lovastatin, a drug used to decrease cholesterol level, and XCT790, an inverse agonist of ERRα, overcome gefitinib and osimertinib resistance both in vitro and in vivo.

**Conclusions:**

Our study indicates that cholesterol/EGFR/Src/Erk/SP1 axis-induced ERRα re-expression promotes survival of gefitinib and osimertinib-resistant cancer cells. Besides, we demonstrate the potential of lowing cholesterol and downregulation of ERRα as effective adjuvant treatment of NSCLC.

**Supplementary Information:**

The online version contains supplementary material available at 10.1186/s12943-022-01547-3.

## Background

Epidermal growth factor receptor (EGFR) is reported as an important driver oncogene in non-small cell lung cancer (NSCLC) [1]. Patients harboring EGFR mutations, including the exon 19 deletion and L858R point mutation, initially respond well to EGFR tyrosine kinase inhibitors (EGFR-TKIs) [2, 3]. However, most patients will eventually develop acquired resistance after 9-12 months of treatment [4, 5]. The most common mechanism of resistance in patients using either the first- or the second-generation EGFR-TKIs is second-site mutation T790M of EGFR exon 20 [6]. The third-generation EGFR-TKIs, including osimertinib and olmutinib, are developed to overcome T790M mutation-mediated resistance [7, 8]. Based on clinical trials, osimertinib become the standard second-line treatment in NSCLC patients harboring T790M mutation [9]. In recent years, osimertinib shows superior overall survival (OS) and progression free survival (PFS) compared with the first-generation EGFR-TKIs (gefitinib or erlotinib) as first-line treatment in NSCLC patients with EGFR mutations [10]. Although osimertinib exhibits promising clinical results, acquired resistance inevitably develops with a median PFS of 19 months in the first-line and 11 months in the second-line treatment [11]. Development of resistance to either the first- or the third-generation EGFR-TKIs limits their therapeutic efficacy. Thus, it is important to identify the drug resistance mechanisms to guide therapeutic regimen.

Reprogramming of lipid metabolism is a hallmark of cancers [12, 13]. As a crucial component of lipids, cholesterol is recognized to be essential for cancer cell proliferation and survival [14]. Apart from being a constituent of cell membrane, cholesterol is widely distributed in lipid rafts, small domains within the cell membrane that represent as platforms involved in cellular signaling transduction [15]. Mammalian cells maintain cholesterol homeostasis through regulation of de novo synthesis, uptake, efflux and storage progress [16]. Most recently, content of cholesterol is found to be upregulated in NSCLC cells or tumor xenograft models that resistant to the first-generation EGFR-TKIs as the result of increased biosynthesis, uptake and receded efflux. Accumulation of cholesterol is regarded as an important factor of resistance to the first-generation EGFR-TKIs [17–19]. However, the mechanisms of cholesterol confer resistance to EGFR-TKIs are not well-revealed.

Estrogen related receptor alpha (ERRα) belongs to the orphan nuclear receptor superfamily and is encoded by the ESRRA gene. As a transcription factor, ERRα is well-known to regulate mitochondrial and metabolic genes involved in cellular energy metabolism [20]. Besides normal metabolism, ERRα exhibits oncogenic functions in various human cancers including lung cancer, breast cancer, prostate cancer and colon cancer [21–23]. The expression of ERRα is upregulated in lung cancer and its overexpression is related to poor survival in patients, suggesting that ERRα can be a druggable target for cancer therapy [24–26]. Moreover, accumulating evidence indicates that intracellular cholesterol is closely linked to the expression and activation of ERRα in breast cancer [27–29]. However, little is known regarding the relationship between cholesterol and ERRα in NSCLC.

In the present study, we investigate cholesterol level in NSCLC and observe that accumulation of cholesterol in lipid rafts correlate to the resistance to both the first- and the third-generation EGFR-TKIs. We identify a regulatory role of cholesterol in ERRα re-expression and elucidate the underlying mechanisms. Mechanistically, we uncover that accumulation of cholesterol in lipid rafts reactivates EGFR/Src/Erk signaling pathway and promotes SP1 nuclear translocation, which enables ERRα transcription in the presence of EGFR-TKIs that is critical to NSCLC resistance.

## Methods

### Reagents

Gefitinib and lovastatin were purchased from Aladdin (Shanghai, China). Osimertinib was obtained from Glpbio (Montclair, CA, USA). XCT790, BAY 11-7082, WH-4-023 and Plicamycin were purchased from MCE (New Jersey, USA). Rapamycin, Y27632 and SCH772984 were obtained from AbMole (Houston, TX, USA). Lapatinib, AG-490 and mevalonate were obtained from Topscience (Shanghai, China)**.** SIS3 was purchased from Selleck (Shanghai, China). Cholesterol and MβCD were obtained from Sigma-Aldrich (St Louis, MO, USA).

### Cell culture

For all experiments, human NSCLC cell line PC-9 (BCRJ, Rio de Janeiro, Brazil), PC-9/GR, H1975 (Cell Bank of the Shanghai Academy of Life Sciences, Shanghai, China), and PC-9/OR were maintained in Dulbecco modified Eagle medium (KGM12800N-500, KeyGen BioTech, Nanjing, China) supplemented with 10% fetal bovine serum (086150, Wisent, St-Bruno, Quebec, Canada),100 μg/mL streptomycin and 100 U/mL penicillin (ST488, Beyotime Biotechnology, Shanghai, China) in a humidified cell culture incubator at 37 °C with an atmosphere of 5% CO_2_.

### Transfection

For cell transfection, 100 nM siEGFR (the target sequence: 5′- GCAACAUGUCGAUGGACUUTT − 3′) (HanBio, Shanghai, China) and 2.5 μg pCDNA3.1 or pCDNA3.1-SP1 (Genomeditech, Shanghai, China) were transfected into NSCLC cells with Lipofectamine 2000 (11668-019, Invitrogen, Carlsbad, CA, USA) growing in serum-free opti-MEM media (51985-034, Gibco, Gaithersburg, MD, USA).

### Western blot

For evaluating protein expression in cells or tumor tissues, proteins were extracted. The same amounts of proteins were separated through 10-15% SDS-PAGE and transferred to polyvinylidene difluoride membranes, following by blocking with 5% nonfat milk. Subsequently, the membranes were incubated with primary antibodies, then with secondary antibodies. Immunoblot signals were visualized by ECL detection and the expression of proteins was quantified by Image J software. Antibodies used for Western blot were: anti-p-EGFR, anti-EGFR, anti-Erk, anti-SP1 (Cell Signaling Technology, Boston, MA, USA); anti-GAPDH, anti-p-Erk (Proteintech, Wuhan, China); anti-p-Src (Affinity Biosciences, Changzhou, China); anti-Lamin B (Wanleibio, Shenyang, China); anti-Caveolin1 (HUABIO, Hangzhou, China); anti-ERRα, anti-Src, HRP-labeled goat anti-rabbit IgG(H + L) (Beyotime Biotechnology, Shanghai, China).

### RT-qPCR

Trizol reagent (R401-01, Vazyme, Nanjing, China) was used to extract total RNAs from NSCLC cells as described by the manufacturer’s instructions. cDNA was synthesized using mRNA as a template with HiScript III RT SuperMix (R323-01, Vazyme, Nanjing, China). ChamQ SYBR qPCR Master Mix (Q341-02, Vazyme, Nanjing, China) was used for RT-qPCR analysis. GAPDH expression was used to normalize the data. The mRNA primer sequences used are shown as below: 5′-GTCTCCTCTGACTTCAACAGCG-3′ and 5′-ACCACCCTGTTGCTGTAGCCAA-3′ for GAPDH; 5′-CCTCTGTGACCTCTTTGACC-3′ and 5′-TACTGACATCTGGTCAGAC-3′ for ERRα [30].

### Cell viability assay

Cell viability was determined by MTT assay. Cells were treated with indicated drugs following by adding 0.5 mg/mL MTT solution (3580GR001, Biofroxx, Guangzhou, China) at 37 °C for 4 h. Finally, the absorbance of each well was measured using a microplate reader.

### Luciferase reporter assay

Cells were transfected with pRL-TK and pGMERRα-Lu (Genomeditech, Shanghai, China) and cultured in medium with indicated drugs for 36 h. Subsequently, cells were lysed with 1× cell lysis buffer at room temperature for 5 min and the luciferase activities were detected with Dual-Luciferase Reporter Assay System (DL101-01, Vazyme, Nanjing, China). Renilla luciferase activity was used to normalize the firefly luciferase activity.

### Co-immunoprecipitation (co-IP)

Cells were treated with indicated drugs and collected, then lysed in RIPA lysis buffer (KGP703-100, KeyGen BioTech, Nanjing, China). Lysates were centrifuged at 14,000 g, 4 °C for 10 min and supernatant were transferred to another tube with EGFR primary antibody (1:100, 4267S, Cell Signaling Technology, Boston, MA, USA) incubated overnight at 4 °C. Then, the protein complexes were collected by incubation with 60 μL of Protein A + G Agarose (P2055, Beyotime Biotechnology, Shanghai, China) for 1 h. Finally, the protein complexes were washed with RIPA lysis buffer and analyzed by Western blot assay.

### CHIP assay

Chromatin immunoprecipitation (CHIP) assay was conducted following the manufacturer’s instructions of the CHIP assay kit (P2078, Beyotime Biotechnology, Shanghai, China). Briefly, H1975 cells were fixed with 1% formaldehyde for 10 min, quenched in glycine for 5 min, washed and lysed in SDS lysis buffer. Cell lysates were sonicated until the DNA fragments were 200-1000 bp in size. Then the lysates were collected and pre-cleared with Protein A + G Agarose/Salmon Sperm DNA for 30 min. 2% percent of the chromatin was used as input control and the rest was incubated with antibody against IgG (1:100, A7016, Beyotime Biotechnology, Shanghai, China) and SP1 (1:100, 9389S, Cell Signaling Technology, Boston, MA, USA) overnight at 4 °C. The chromatin complexes were then incubated with Protein A + G Agarose/Salmon Sperm DNA for 1 h at 4 °C, following by washed with low salt immune complex wash buffer, high salt immune complex wash buffer, LiCl immune complex wash buffer, TE buffer and elution buffer (1% SDS, 0.1 M NaHCO_3_). The DNA-protein complexes were incubated with 0.2 M NaCl overnight at 65 °C, RNase A for 30 min at 37 °C, proteinase K (ST532, Beyotime Biotechnology, Shanghai, China) for 1.5 h at 45 °C. The DNA was purified using a DNA Clean Up Kit (D0033, Beyotime Biotechnology, Shanghai, China). Finally, the precipitated DNA was quantified using qPCR. Primers used for ERRα were: 5′-TGACCCCATCCGAGTGGAATTTGAGT-3′ and 5′-AGAAAGCTCAAGGTCACTGCGGTG for-3′.

### Immunofluorescence

For EGFR and Src fluorescence colocalization analysis, cells were seeded and exposed to the indicated drugs. Then, cells were fixed with 4% paraformaldehyde, blocked with goat serum and incubated with primary antibody against EGFR (1:100, 4267S, Cell Signaling Technology, Boston, MA, USA) overnight at 4 °C. CoraLite®594-conjugated c-SRC (1:100, CL594-60315, Proteintech, Wuhan, China) and Goat Anti-Rabbit IgG H&L (FITC) (1:100, ab97050, Abcam, Cambridge, MA, USA) were used to treated the cells for 2 h at 37 °C. nucleus was counterstained with 5 μg/mL DAPI (C1002, Beyotime Biotechnology, Shanghai, China) for 10 min at room temperature. All images were taken with Zeiss LSM800 and measured by Image Pro Plus software [31]. Pearson’s correlation coefficient was calculated by Image J software [32, 33].

### IHC

One-Step IHC Assay kit (KGOS60, KeyGen BioTech, Nanjing, China) was used for immunohistochemical (IHC) staining. Tissue slides acquired from NSCLC xenografts were deparaffinized and rehydrated, following by antigen retrieval, permeabilizated and 3% H_2_O_2_ treatment. After being blocked with goat serum, slides were incubated with primary antibody against Ki67 (1:100, WL01384a, Wanleibio, Shenyang, China) or ERRα (1:100, sc-65715, Santa Cruz Biotechnology, Dallas, TX, USA) overnight at 4 °C. On the second day, immune complexes were determined with horseradish peroxidase conjugates using DAB detection.

### Nuclear protein extraction

The nuclear protein was extracted using Nuclear and Cytoplasmic Protein Extraction Kit (P0027, Beyotime Biotechnology, Shanghai, China) according to the manufacturer’s instructions. Briefly, cells that have been treated with indicated drugs were harvested and dissociated in 200 μL Reagent A mixture containing 1 mM PMSF (KGP610, KeyGen BioTech, Nanjing, China). The cell suspension was incubated on ice for 15 min. Then, 10 μL Reagent B was added and the mixture was vortexed for 5 s with 1 min ice bath, following by centrifugation at 16,000 g, 4 °C for 5 min. The supernatant was collected as cytoplasmic protein and the precipitation was further resuspended in 50 μL nuclear protein extraction reagent containing 1 mM PMSF. After being vortexed and ice bath in turn for 30 min, the mixture was centrifugated at 16,000 g, 4 °C for 5 min and the supernatant was saved as nuclear protein. The subcellular fractions were analyzed by Western blot assay.

### Isolation of lipid rafts [34]

Cells were seeded, treated with indicated drugs and collected. The lysis buffer (20 mM Tris-HCl (pH 7.5), 150 mM NaCl, 0.5% Triton X-100) was used to lyse cells for 30 min. Then, the lysates were centrifugated at 16,000 g, 4 °C for 30 min and supernatants were collected and referred to as the non-lipid rafts fractions. The rest insoluble pellets were added with 200 μL buffer (0.5% SDS and 2 mM DTT), re-suspended and incubated for 10 min. Finally, the samples were centrifugated at 16,000 g, 4 °C for 30 min and the supernatants were collected into another tubes, referring to as lipid rafts fractions.

### Tumor xenograft model

BALB/c nude mice (4 weeks old) (Yangzhou University, Yangzhou, China) were housed under individual ventilated cages in compliance with the institutional guidelines. Mice were randomly assigned into four groups (*n* = 5), injected with PC-9, PC-9/GR, H1975 and PC-9/OR cells subcutaneously. We monitored the tumor volume every 3 days. In the second xenograft experiments, PC-9/GR cells were subcutaneously injected into the mice. After 1 week injection, mice were randomly assigned into three groups (*n* = 5) according to tumor volume as follows: gefitinib group (treat with 50 mg/kg gefitinib), gefitinib + lovastatin group (treat with 50 mg/kg gefitinib and 100 mg/kg lovastatin), and gefitinib + XCT790 group (treat with 50 mg/kg gefitinib and 8 mg/kg XCT790). In all experiments, gefitinib and lovastatin were administrated by oral gavage daily, while XCT790 was administrated by intraperitoneal injection. The tumor volume was measured every 3 days and calculated based on the formula: tumor volume (mm^3^) = (length×width^2^)/2. After approximately 3 weeks, mice were sacrificed and xenografts tissue were collected for further analysis.

### Statistical analysis

Data were presented as mean ± SEM and statistical analysis were performed by GraphPad Prism 8.0 software (GraphPad Software Inc., CA, USA). Unpaired Student’s t test was used to compare differences between two groups, while analysis of variance (ANOVA) was used for comparisons among multiple groups. The Kaplan–Meier method was used for survival analysis, and the log-rank test was used to determine the statistical significance. *P* < 0.05 was considered statistically significant.

## Results

### Generation of gefitinib-resistant and osimertinib-resistant NSCLC cells

**Fig. 1:**
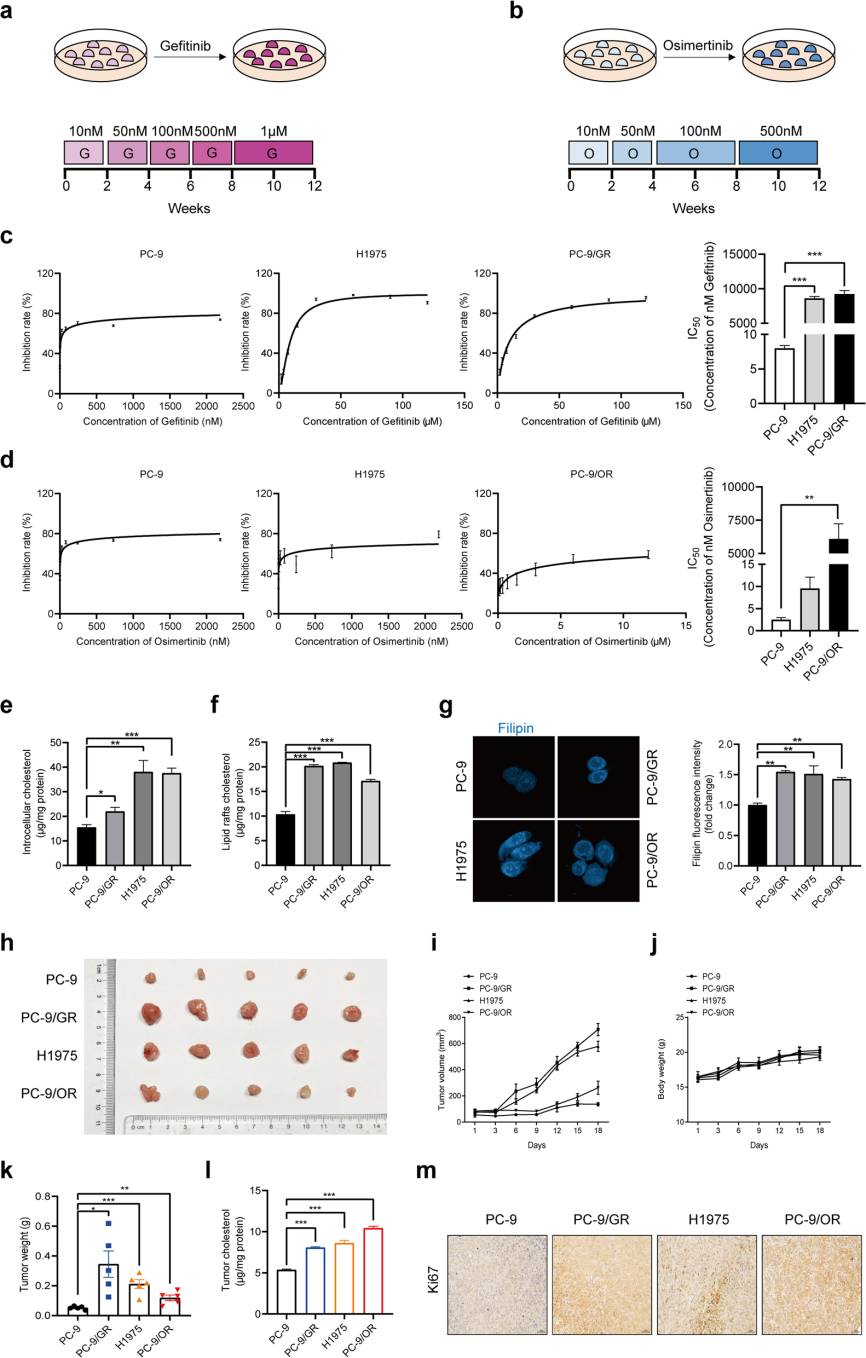
Accumulation of cholesterol in lipid rafts is responsible for EGFR-TKIs resistance. **a**, **b** PC-9 cells were exposed to gefitinib or osimertinib for 12 weeks to establish the gefitinib-resistant PC-9/GR and osimertinib-resistant PC-9/OR cells. **c**, **d** MTT assay was conducted to detect cell viability of NSCLC cells treated with gefitinib or osimertinib. **e**, **f** An Amplex® Red Cholesterol Assay Kit (A12216, Invitrogen, Carlsbad, CA, USA) was used to determine cholesterol level in the cytoplasm or lipid rafts of NSCLC cells. **g** Cells were incubated with 0.5 mg/mL Filipin (MB1848, Meilunbio, Dalian, China) for 2 h. Confocal images showed the free cholesterol in blue and the fluorescence intensity was analyzed. **h-m** Primary tumor gross appearance, **i** growth curves, **j** body weight, and **k** tumor weight of the tumor xenograft. **l** Cholesterol level in tumor tissues was determined. **m** IHC staining detected Ki67 expression in the indicated tumors. Data are expressed as mean ± SEM (*n* = 3) *p < 0.05, **p < 0.01, ***p < 0.001 in **c**, **d**, **e**, **f**, **g** and **l**. Data are expressed as mean ± SEM (*n* = 5) *p < 0.05, **p < 0.01, ***p < 0.001 in **k**

To generate gefitinib-resistant PC-9/GR and osimertinib-resistant PC-9/OR cells, parental PC-9 cells were exposed to increasing concentrations of gefitinib or osimertinib starting at 10 nM, until they were able to freely proliferate in 1 μM gefitinib or 500 nM osimertinib, which occurred after 12 weeks of drug treatment (Fig. 1a, b). H1975 cells, harboring EGFR T790M mutation, were used in our study. MTT assay confirmed that cell viability of PC-9 cells was markedly inhibited by gefitinib or osimertinib compared to PC-9/GR, H1975 or PC-9/OR cells (Fig. 1c, d).

### Accumulation of cholesterol promotes EGFR-TKIs resistance

Our previous studies have found that intracellular cholesterol in gefitinib-resistant NSCLC cells was higher than that in sensitive cells and was involved in gefitinib resistance [19]. However, the role of cholesterol in osimertinib resistance and mechanisms leading to the resistance have not been fully revealed. We therefore evaluated the total cholesterol and lipid rafts cholesterol level by an Amplex® Red Cholesterol Assay Kit, while measured free cholesterol by filipin staining. All three types of cholesterol exhibited comparable increases in PC-9/GR, H1975, PC-9/OR cells, indicating that cholesterol accumulation occurred during NSCLC obtained gefitinib and osimertinib tolerance (Fig. 1e-g). In several studies, MβCD was used to remove cholesterol and explore the influence of cholesterol on cell viability in vitro. Our team have also done these experiments and found MβCD could sensitize NSCLC cells to EGFR-TKIs treatment. In this study, we further explored the biological function of cholesterol in the progression of gefitinib and osimertinib resistance via xenograft model. It was found that PC-9/GR, H1975, PC-9/OR xenograft tumors were larger than PC-9 xenograft counterparts both in size and weight and contained more cholesterol in tumor tissues (Fig. 1h-l). In addition, Ki67 expression was upregulated, suggesting that accumulation of cholesterol enhanced the proliferation ability of NSCLC, which might contribute to the progression of EGFR-TKIs resistance (Fig. 1m). These results revealed that accumulation of cholesterol-induced cell proliferation was responsible for both gefitinib and osimertinib resistance.

### Re-expression of ERRα contributes to EGFR-TKIs resistance

**Fig. 2:**
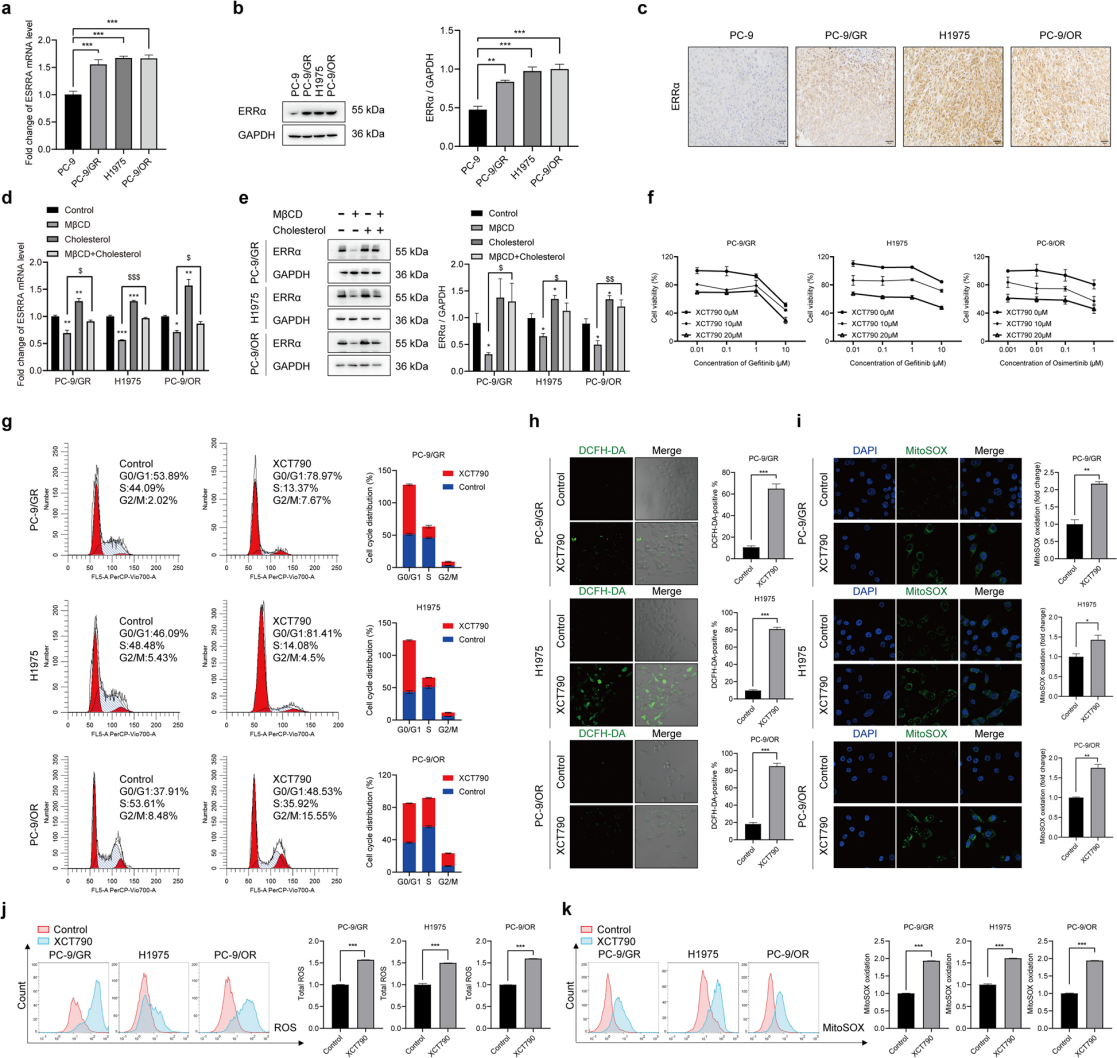
Cholesterol-mediated ERRα overexpression is responsible for EGFR-TKIs resistance. **a**, **b** Results of RT-qPCR and Western blot assay showed ERRα mRNA and protein level in indicated cells. **c** IHC staining detected ERRα expression in tumors. **d** Cells were treated with 2.5 mM MβCD, 10 µM cholesterol or 2.5 mM MβCD +10 µM cholesterol for 6h, then ERRα mRNA level in indicated cells was determined by RT-qPCR assay. **e** Cells were treated with 2.5 mM MβCD, 10 µM cholesterol or 2.5 mM MβCD +10 µM cholesterol for 24h, then ERRα protein level in indicated cells was determined Western blot assay. **f** Cells were treated with gefitinib, osimertinib alone or combined with XCT790 for 48 h. Then MTT assay was conducted. **g** Cells were cultured in medium with XCT790 for 48 h, then fixed with cold ethanol overnight, incubated with PI/RNase staining buffer (550825, BD, Franklin Lake, NJ, USA) for 15 min. Cell cycle distribution was analyzed by flow cytometry. **h** Cells were treated with XCT790 for 48 h and then incubated with DCFH-DA (S0033S, Beyotime Biotechnology, Shanghai, China) for 30 min. Confocal images showed the ROS in green and the fluorescence intensity was analyzed. **i** Cells were treated with XCT790 for 48 h and then fixed and incubated with 5 μM MitoSOX Red Mitochondrial Superoxide Indicator (40778ES50, Yeasen, Shanghai, China) for 10 min. Confocal images showed the MitoSOX in green and the fluorescence intensity was analyzed. **j**, **k** Flow cytometry analysis was conducted to determine the ROS and MitoSOX accumulation in indicated cells. Data are expressed as mean ± SEM (*n* = 3) *p < 0.05, **p < 0.01, ***p < 0.001 in **a**, **b**, **h**, **i**, **j** and **k**. Data are expressed as mean ± SEM (*n* = 3) *p < 0.05, **p < 0.01, ***p < 0.001 compared to control; ^$^*p* < 0.05, ^$$^*p* < 0.01, ^$$$^*p* < 0.001 compared to MβCD in **d** and **e**

To explore the underlying mechanisms of cholesterol in promoting EGFR-TKIs resistance, we determined the expression of ERRα, a nuclear receptor connected to cholesterol-mediated signal transduction [27, 35]. Firstly, we searched the OncoMine database (https://www.oncomine.org/resource/login.html) and found ERRα was significantly increased in lung adenocarcinoma (Fig. S1a). Kaplan-Meier survival plot showed that lower expression of ERRα resulted in a better survival (Fig. S1b). Next, the result of RT-qPCR, Western blot and IHC assays furtherly revealed that ERRα mRNA and protein level was significantly upregulated in PC-9/GR, H1975, PC-9/OR cells and xenograft tumors compared with PC-9 cells, suggesting enhanced ERRα expression was related to NSCLC progression and development of EGFR-TKIs resistance (Fig. 2a-c). Then, we explored the relationship between changed cholesterol level and ERRα expression. It was found that MβCD decreased ERRα expression and cholesterol supplement eliminated the effect of MβCD (Fig. 2d, e). These data indicated that accumulation of cholesterol conferred NSCLC resistance to EGFR-TKIs via upregulating ERRα expression.

Subsequently, we explored the contribution of ERRα in acquired resistance to EGFR-TKIs. XCT790, an inverse agonist of ERRα, was used to downregulate the expression of ERRα. As shown, XCT790 sensitized PC-9/GR, H1975 cells to gefitinib and PC-9/OR cells to osimertinib significantly (Fig. 2f). Moreover, XCT790 induced G0/G1 phase arrest, increased total ROS and mitochondria ROS superoxide radicals in NSCLC cells (Fig. 2g-k). These data suggested that downregulation of ERRα expression sensitized resistant cells to EGFR-TKIs via increasing ROS level and causing cell cycle arrest in G0/G1 phase.

### ERRα re-expression is regulated by EGFR activation

**Fig. 3:**
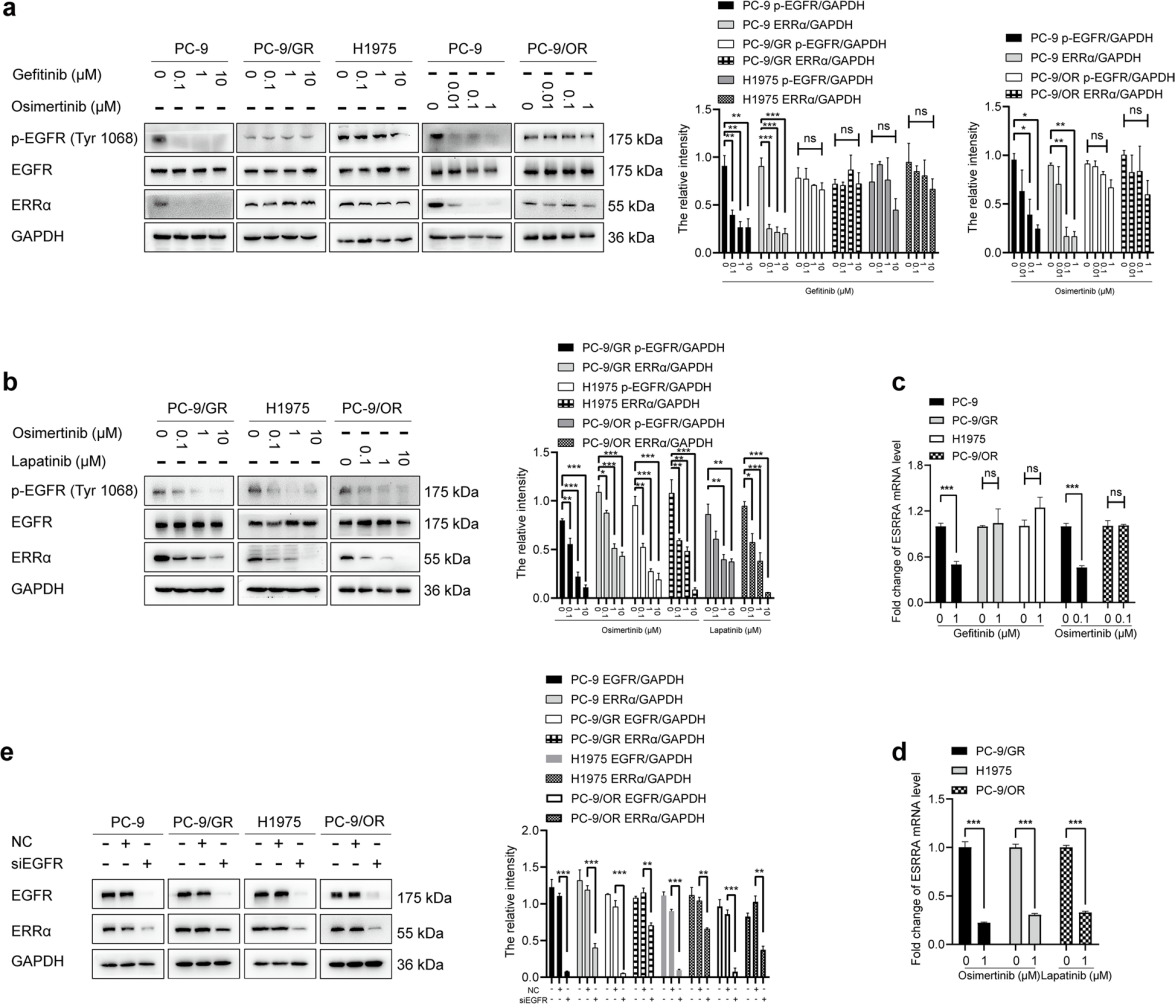
EGFR activation sustains ERRα re-expression. **a** Western blot assay showed p-EGFR, EGFR and ERRα proteins level after treatment with gefitinib or osimertinib. **b** Western blot assay showed the proteins level after treatment with osimertinib or lapatinib. **c** PC-9, PC-9/GR, H1975 and PC-9/OR cells were treated with 1 μM gefitinib or 0.1 μM osimertinib for 6 h. RT-qPCR showed ERRα mRNA level. **d** PC-9/GR, H1975, PC-9/OR cells were treated with 1 μM osimertinib or 1 μM lapatinib for 6 h. RT-qPCR showed ERRα mRNA level. **e** Cells were transfected with NC or siEGFR, after 48 h, cells were harvested and the proteins level were detected by Western blot assay. Data are expressed as mean ± SEM (*n* = 3) *p < 0.05, **p < 0.01, ***p < 0.001 in **a**, **b**, **c**, **d** and **e**

It has been reported that cholesterol influenced EGFR activation via changing the localization of EGFR on cell membrane [36]. To further investigate the signaling pathways that regulates ERRα expression during NSCLC developed drug resistance, we used EGFR-TKIs to treat NSCLC cells. It was found that gefitinib failed to decrease p-EGFR level in PC-9/GR and H1975 cells. Similarly, osimertinib failed to reduce p-EGFR level in PC-9/OR cells. However, the expression of p-EGFR was significantly depleted by gefitinib or osimertinib in sensitive PC-9 cells. Then, ERRα expression was measured under the same treatment. Notably, gefitinib or osimertinib inhibited ERRα expression in PC-9 cells. No significant changes of ERRα expression were observed in PC-9/GR, H1975, and PC-9/OR cells (Fig. 3a). To identify whether ablation of p-EGFR could then decrease ERRα expression, we reduced p-EGFR level by osimertinib in PC-9/GR and H1975 cells, and lapatinib in PC-9/OR cells. Results showed that p-EGFR inhibition led to decreased ERRα expression (Fig. 3b). Similar consequence was observed in ERRα mRNA level (Fig. 3c, d). To rule out that the decrease of ERRα expression was caused by cell death, we treated all the four cell lines with cisplatin and noticed that cisplatin failed to alter ERRα expression (Fig. S2a). In order to exclude the off-target effects of EGFR-TKIs, we silence EGFR by siRNA. As expected, ERRα expression was downregulated by EGFR silence in NSCLC cells (Fig. 3e). These data indicated the positive regulation of ERRα expression by EGFR signaling activation in the progress of TKIs resistance.

### Cholesterol-mediated EGFR/Src/Erk signaling activation sustains ERRα re-expression

**Fig. 4:**
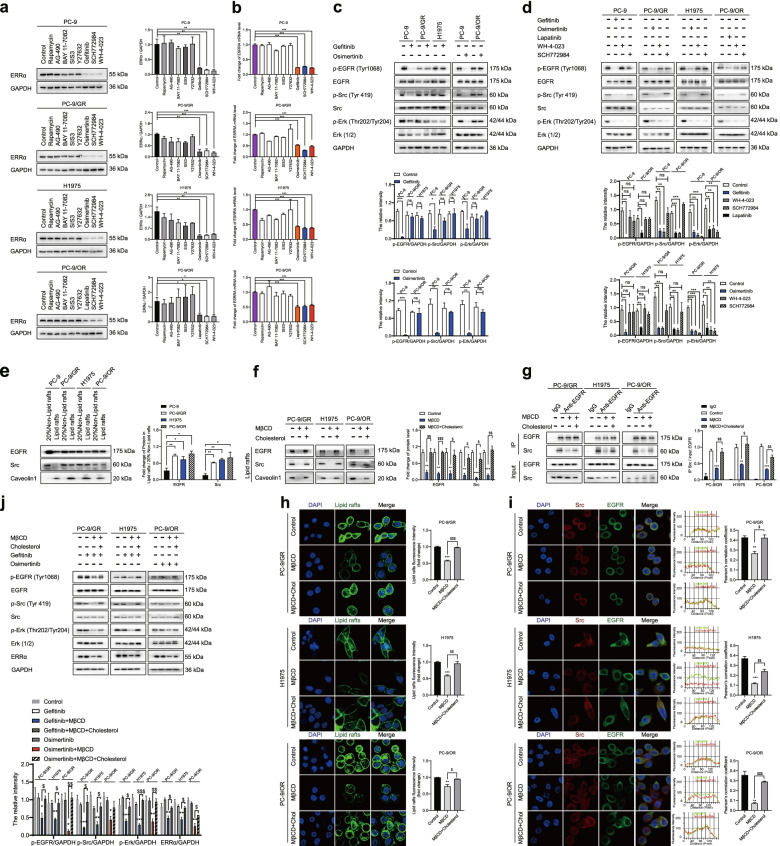
Cholesterol-induced EGFR/Src/Erk signaling reactivation sustains ERRα re-expression. **a, b** Western blot and RT-qPCR assay showed ERRα expression after treatment with inhibitors of mTOR, STAT3, NF-κB, Smad3, ROCK, EGFR, Erk or Src. **c** Cells were treated with 1 μM gefitinib or 0.1 μM osimertinib for 48 h, Western blot assay showed the proteins level. **d** Cells were treated with gefitinib, osimertinib, lapatinib, WH-4-023 or SCH772984 for 48 h, Western blot assay showed the proteins level. **e** Proteins in lipid rafts was extracted. Western blot assay showed EGFR and Src protein level in 20% non-lipid rafts fractions and lipid rafts fractions. **f** Cells were harvested after treatment with 10 mM MβCD for 45 min or 10 mM MβCD for 45 min + 10 μM cholesterol for another 2 h. Proteins in lipid rafts were extracted and EGFR, Src, Caveolin1 expression was detected. **g** Co-immunoprecipitation analysis showed the EGFR and Src interaction. **h** Cells were incubated with 1 μg/mL CTB for 1 h after treatment with MβCD or MβCD+ cholesterol. Confocal images showed the lipid rafts in green and the fluorescence intensity was analyzed. **i** Immunofluorescence staining of EGFR (green) and Src (red) markers in the indicated NSCLC cells after treatment with MβCD or MβCD + cholesterol. **j** Cells were treated with MβCD or MβCD + cholesterol then subsequently with gefitinib or osimertinib for 48 h, proteins levels were detected. Data are expressed as mean ± SEM (*n* = 3) *p < 0.05, **p < 0.01, ***p < 0.001 in **a**, **b**, **c**, **d** and **e**. Data are expressed as mean ± SEM (*n* = 3) **p < 0.01, ***p < 0.001 compared to control; ^$^*p* < 0.05, ^$$^*p* < 0.01, ^$$$^*p* < 0.001 compared to MβCD in **f**, **g**, **h** and **i**. Data are expressed as mean ± SEM (*n* = 3) *p < 0.05, **p < 0.01, ***p < 0.001 compared to gefitinib or osimertinib; ^$^*p* < 0.05, ^$$^*p* < 0.01, ^$$$^*p* < 0.001 compared to gefitinib + MβCD or osimertinib + MβCD in **j**

To determine which signaling pathway drives the re-expression of ERRα after EGFR signaling activation, the most commonly aberrantly activated pathways that include mTOR, STAT3, NF-κB, Smad, ROCK, Erk and Src were tested by inhibitors rapamycin, AG-490, BAY 11-7082, SIS3, Y27632, SCH772984 and WH-4-023, respectively. We identified that the kinase inhibitors for Src and Erk consistently suppressed ERRα protein and mRNA expression in NSCLC cells (Fig. 4a, b). Further analysis showed that EGFR, Src and Erk signaling is insensitive to the inhibitory action of gefitinib or osimertinib in drug resistant cells (Fig. 4c). To dissect the regulatory hierarchy, inhibitors of EGFR, Src and Erk were used to treat NSCLC cells, respectively. As shown, p-Src and p-Erk were inhibited after EGFR signaling being blocked. In addition, WH-4-023 downregulated p-Erk expression while SCH772984 had no influence on p-Src level (Fig. 4d). These data implied that reactivation of EGFR/Src/Erk cascade sustained the re-expression of ERRα in EGFR-TKIs resistant NSCLC cells.

Next, we explored the reason by which caused EGFR/Src/Erk cascade reactivation during NSCLC resistance to EGFR-TKIs. EGFR subcellular localization is important for the activation of its downstream signaling molecules [37]. We isolated lipid rafts and non-lipid rafts fractions of NSCLC cells and found that the localization of EGFR as well as Src to lipid rafts fractions was most prominent in the EGFR-TKIs resistant cells compared to sensitive cells (Fig. 4e). When removed cholesterol by MβCD, localization of EGFR and Src to lipid rafts fractions was decreased. However, cholesterol supplement eliminated the effect of MβCD, EGFR and Src re-localized in lipid rafts (Fig. 4f). To determine whether EGFR co-localized with Src, cells were coimmunostained with EGFR and Src antibodies. It was found that co-localization between EGFR and Src occurred along the membrane of NSCLC cells. In addition, MβCD depleted the colocalization but cholesterol restored (Fig. 4h, i). Consistently, MβCD weaken physical interaction between EGFR and Src, but cholesterol restored (Fig. 4g). Moreover, activation of EGFR/Src/Erk signaling and expression of ERRα were inhibited by gefitinib or osimertinib when MβCD was added in resistant cells. Whereas, the effect of MβCD was offset by cholesterol (Fig. 4j). These results illustrated that accumulation of cholesterol in lipid rafts during EGFR-TKIs resistance provided a platform for EGFR and Src interaction so as to activate EGFR/Src/Erk signaling pathway and finally promote ERRα expression.

### The cholesterol/EGFR/Src/Erk axis downstream molecule SP1 directly promotes transcription of ERRα

**Fig. 5:**
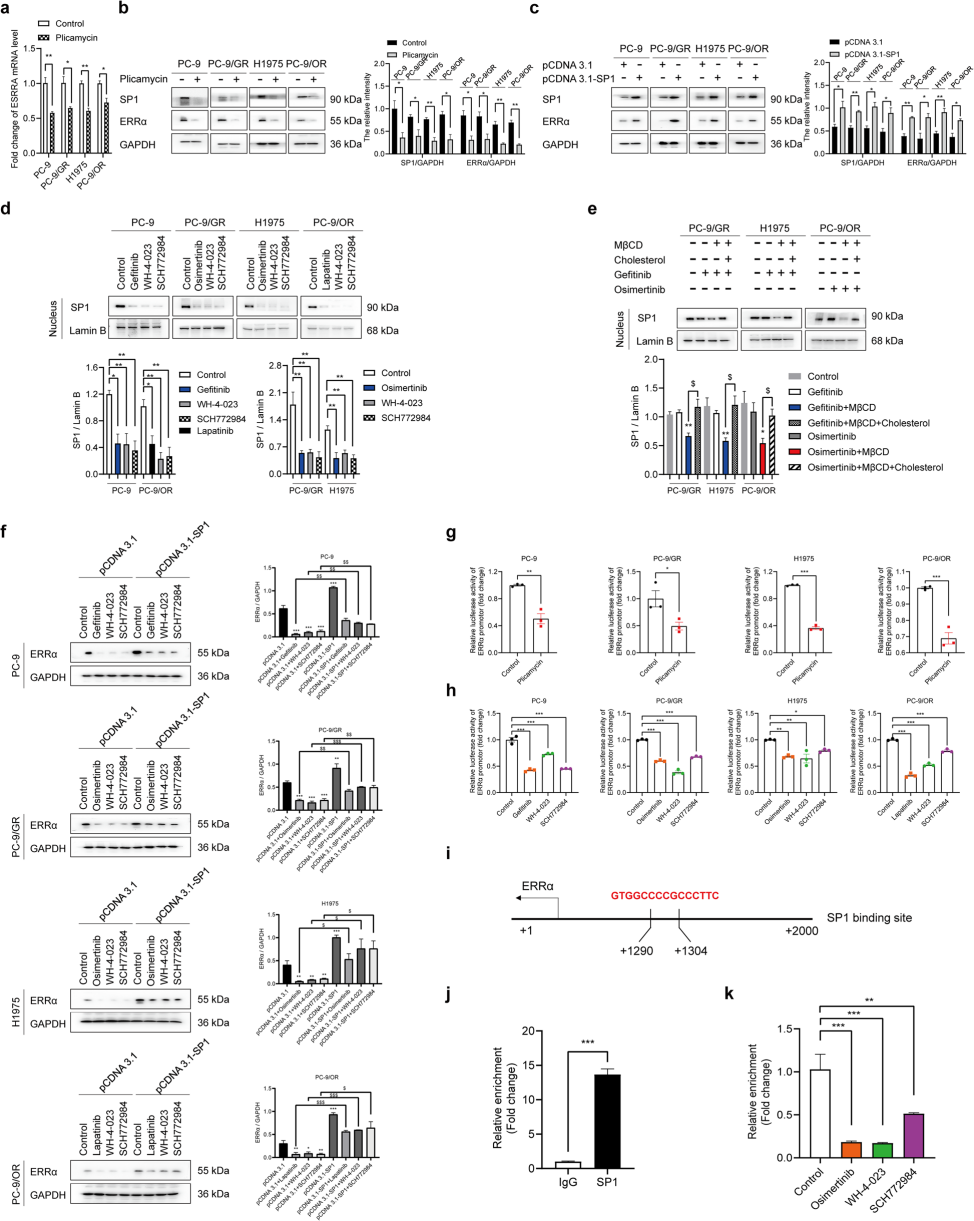
SP1 transcribes ERRα directly and cholesterol/EGFR/Src/Erk axis regulates SP1 nuclear translocation. **a**, **b** RT-qPCR and Western blot assay showed SP1 or ERRα protein and mRNA level after treatment with SP1 inhibitor (plicamycin). **c** Cells were transfected with empty pCDNA3.1 or pCDNA3.1-SP1 for 48 h. The protein levels of SP1 and ERRα were determined by Western blot assay. **d** Cells were harvested after treatment with inhibitors of EGFR (gefitinib or osimertinib or lapatinib), Erk (SCH772984), Src (WH-4-023) for 48 h. Proteins in nucleus were extracted and SP1 expression was conducted by Western blot assay. **e** Cells were harvested after treatment with 10 mM MβCD for 45 min or 10 mM MβCD for 45 min + 10 μM cholesterol for another 2 h, then with 1 μM gefitinib or 0.1 μM osimertinib for 48 h. Proteins in nucleus were extracted and SP1 expression was conducted by Western blot assay. **f** Cells were transfected with empty pCDNA3.1 or pCDNA3.1-SP1 along with inhibitors of EGFR, Erk, Src for 48 h. The protein levels of ERRα were determined by Western blot assay. **g**, **h** Luciferase assay was performed to determine ERRα promotor activity. **i** Binding site of SP1 was at the promoter region − 1304 to − 1290 bp. **j** CHIP-qPCR analysis showed that the promoter amplicons in the SP1-binding site precipitated by anti-SP1 antibody and anti-IgG antibody in H1975 cells. **k** CHIP-qPCR analysis was performed after cells treated with osimertinib, SCH772984, WH-4-023 for 48 h in H1975 cells. Data are expressed as mean ± SEM (*n* = 3) *p < 0.05, **p < 0.01, ***p < 0.001 in **a**, **b**, **c**, **d**, **g**, **h**, **j** and **k**. Data are expressed as mean ± SEM (*n* = 3) *p < 0.05, **p < 0.01 compared to gefitinib or osimertinib; ^$^*p* < 0.05 compared to gefitinib + MβCD or osimertinib + MβCD in **e**. Data are expressed as mean ± SEM (*n* = 3) *p < 0.05, **p < 0.01, ***p < 0.001 compared to pCDNA3.1; ^$^*p* < 0.05, ^$$^*p* < 0.01, ^$$$^*p* < 0.001 compared to pCDNA3.1 + inhibitors in **f**

Since ERRα expression was regulated by cholesterol/EGFR/Src/Erk signaling cascade, we wondered how the regulatory effect occurred. Cycloheximide (CHX) chase assay was used to evaluate the function of EGFR-TKIs on ERRα protein stability and no significant influence was observed (Fig. S2b). However, as mentioned before, blocking EGFR/Src/Erk signaling activation reduced mRNA level of ERRα. We speculated that the regulation might occur at the transcriptional level and the prediction of transcription factors was performed by UCSC Genome Browser Database (http://genome.ucsc.edu/). Seven potential transcription factors that regulate ERRα including CTCFL, PLAG1, CTCF, SP1, SP2, KLF16 and NRF1 were identified (Table S1). Next, we used JASPAR database (http://jaspar.genereg.net/) to predict transcription factor binding sites. The result demonstrated that SP1 bound to ERRα promoter region with the highest score and with the largest number of transcription factor binding sites (Tables S2, S3), which suggesting SP1 as the most possible transcription factor that regulate ERRα. In consistent with the consequence of prediction, we found that reduction of SP1 expression by plicamycin decreased ERRα expression in both mRNA and protein levels (Fig. 5a, b). The overexpression of SP1 enhanced ERRα expression (Fig. 5c). Meanwhile, inhibitors of EGFR/Src/Erk signaling suppressed nuclear translocation of SP1 (Fig. 5d). In addition, elimination of cholesterol by MβCD treatment promoted gefitinib or osimertinib to restrain SP1 nuclear translocation. The effect was weakened by cholesterol supplement (Fig. 5e). Furthermore, overexpression of SP1 blocked the inhibitory effect of EGFR/Src/Erk inhibitors on ERRα expression (Fig. 5f).

We performed a luciferase assay and found that ERRα promoter activities were downregulated by plicamycin, gefitinib or osimertinib or lapatinib, WH-4-023 and SCH772984 in NSCLC cells (Fig. 5g, h). CHIP assay showed that compared with anti-IgG antibody, at least a 12-fold enrichment of the promoter of ERRα-binding site immunoprecipitated by anti-SP1 antibody was detected, suggesting that SP1 could directly bind to the promoter region (at − 1304 to -1290 bp) of ERRα (Fig. 5i, j). Inhibition of EGFR/Src/Erk cascade could reduce the binding of SP1 and ERRα promoter (Fig. 5k). These results indicated that cholesterol-mediated EGFR/Src/Erk signaling activation could increase SP1 nuclear translocation, enabling ERRα transcription by directly binding to its promoter.

### Combination of EGFR-TKIs with lovastatin or XCT790 overcome NSCLC resistance

**Fig. 6:**
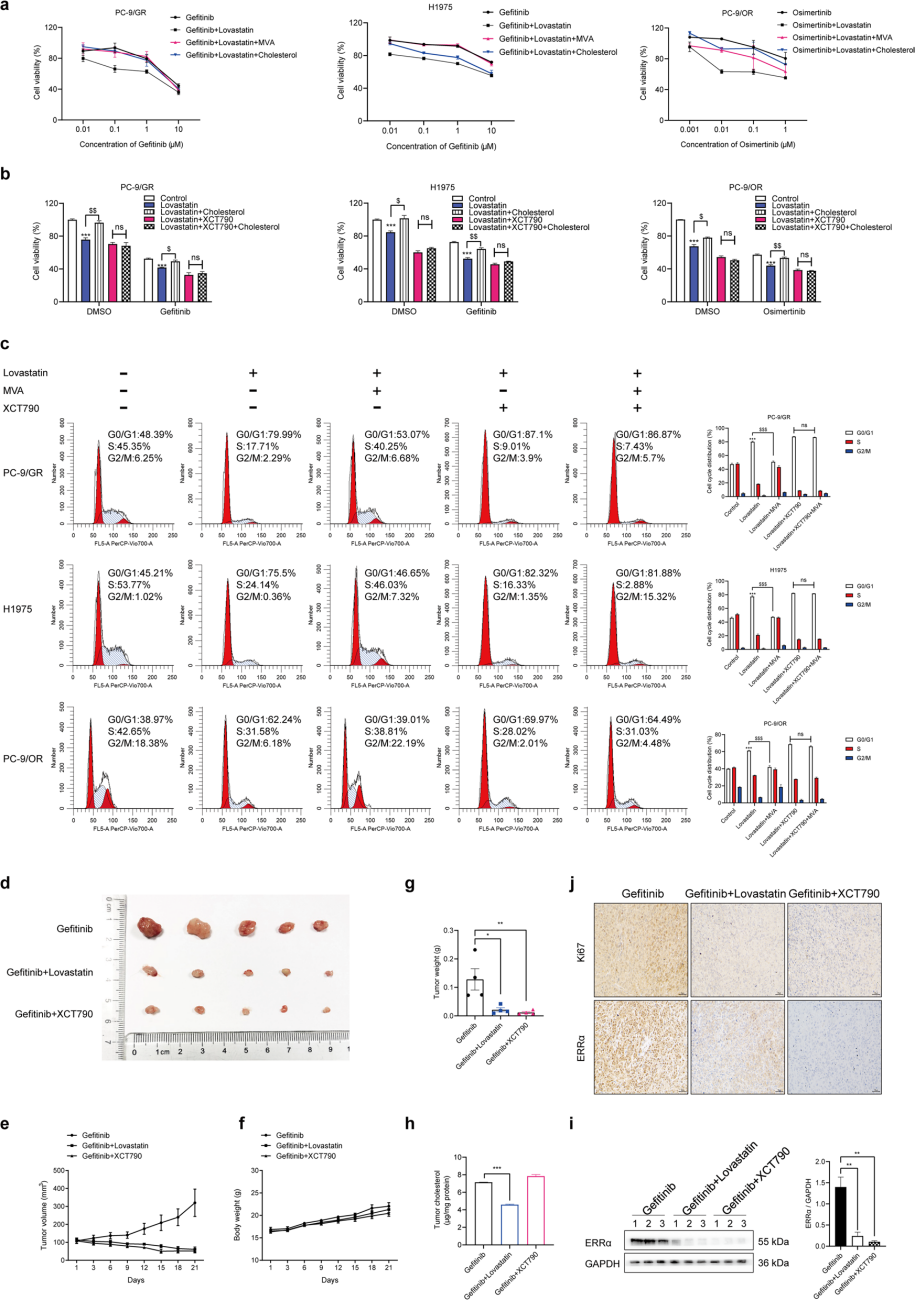
Lovastatin and ERRα inverse agonist enhance the sensitivity of EGFR-TKIs. **a** Cells were treated with gefitinib or osimertinib, gefitinib or osimertinib+ 10 μM lovastatin, gefitinib or osimertinib+ 10 μM lovastatin+ 100 μM mevalonate, gefitinib or osimertinib+ 10 μM lovastatin+ 10 μM cholesterol for 48 h. Then cell viability was measured by MTT assay. **b** Cells were treated with gefitinib or osimertinib, along with lovastatin, lovastatin+ cholesterol, lovastatin+ XCT790, lovastatin+ cholesterol+ XCT790. Then cell viability was measured by MTT assay. **c** Cell cycle distribution was analyzed by flow cytometry after treatment with lovastatin, lovastatin+ MVA, lovastatin+ XCT790, lovastatin+ MVA+ XCT790. **d-j** Primary tumor gross appearance, **e** growth curves, **f** body weight, and **g** tumor weight of the PC-9/GR xenograft after treatment with indicated drugs for 21 days. **h** Cholesterol level in tumor tissue was determined. **i** Western blot assay measured ERRα protein level. **j** IHC staining detected Ki67 and ERRα expression in the indicated tumors. Data are expressed as mean ± SEM (*n* = 3) *p < 0.05, **p < 0.01, ***p < 0.001 compared to control; ^$^*p* < 0.05, ^$$^*p* < 0.01, ^$$$^*p* < 0.001 compared to lovastatin in **b** and **c**. Data are expressed as mean ± SEM (*n* = 3) **p < 0.01, ***p < 0.001in **h** and **i**. Data are expressed as mean ± SEM (*n* = 5) *p < 0.05, **p < 0.01 in **g**

Given the involvement of cholesterol/EGFR/Src/Erk/SP1/ERRα axis in the EGFR-TKIs resistance, we tested whether lowing cholesterol in lipid rafts and inhibiting ERRα expression could affect the sensitivity of EGFR-TKIs in NSCLC. Pharmacological lowing cholesterol using lovastatin combined with gefitinib or osimertinib lead to a significant decrease in proliferation of the drug resistant cells compared to using EGFR-TKIs alone, and the effect of lovastatin was counteracted by mevalonate or cholesterol replenishment (Fig. 6a). To investigate whether the cholesterol pathway and ERRα are functionally dependent, the drug resistant cells were treated with lovastatin and the results showed that the cells were re-sensitized to gefitinib or osimertinib, cell proliferation was suppressed and cell cycle was arrested at the G0/G1 phase. Treatment with the inverse agonist of ERRα XCT790 exhibited similar effects. The effects of lovastatin were largely rescued by cholesterol or mevalonate addition, but this rescue was absent when ERRα expression was downregulated by XCT790, suggesting that the sensitizing effect of lovastatin on NSCLC EGFR-TKIs resistant cells are ERRα-dependent in vitro (Fig. 6b, c). The in vivo experiments confirmed that lovastatin or XCT790 synergized with gefitinib to inhibit xenograft tumor growth (Fig. 6d-j).

## Discussion

Gefitinib belongs to the first-generation EGFR-TKIs and represents a major advance in the treatment of NSCLC harboring EGFR activating mutations. After treatment for about 1 year, drug resistance arises and the most frequently identified drug resistant mechanism is secondary T790M mutation of EGFR [38]. The third-generation TKI, osimertinib is designed to overcome EGFR T790M mutation and brings tremendous success as the second-line use. Osimertinib is found to be superior to gefitinib for the treatment of NSCLC patients with EGFR mutations and used as the first-line therapy in clinical trials. However, drug resistance developed inevitably. Both the first- and the third-generation EGFR-TKIs developed resistance and the mechanisms are not well understood [39]. To better understand the reason why resistance happened, we exposed human PC-9 cells to gefitinib and osimertinib for 12 weeks to establish gefitinib-resistant PC-9/GR cells and osimertinib-resistant PC-9/OR cells. H1975 cells harboring T790M mutation were also used as a gefitinib-resistant model in this study.

Cancer cells require a large amount of lipids to maintain biological membranes and sustain its characteristic of uncontrolled proliferation [40]. Cholesterol as an important component of lipids contribute to rapid cancer cell growth, metastasis and drug resistance [41]. Recently, the alteration of cholesterol homeostasis was reported in NSCLC as a result of dysregulation in cholesterol synthesis, uptake or trafficking process [17, 42]. In the present study, we found upregulated cholesterol level in both gefitinib-resistant PC-9/GR, H1975 cells and osimertinib-resistant PC-9/OR cells. The increase of cholesterol including intracellular total cholesterol and free cholesterol. Lipid rafts were membrane domains that enrich phospholipids, cholesterol and fatty acyl chains [43]. Pro-oncogenic proteins, such as Src, SMO and c-MET, localize in lipid rafts and initiate signaling transduction, indicating potential involvement of lipid rafts in cancer progression [44–46]. In this work, we observed that cholesterol level in lipid rafts of resistant cells was also increased. In xenograft tumor model, PC-9/GR, H1975, PC-9/OR showed faster growth rate and increased Ki67 expression when compared to PC-9, indicating that accumulation of cholesterol promoted growth of NSCLC.

How does cholesterol confer NSCLC cells resistant to EGFR-TKIs? We detected ERRα expression and found that compared to PC-9 cells, PC-9/GR, H1975, PC-9/OR cells showed higher mRNA and protein level of ERRα. ERRα is a nuclear transcription factor and a key regulator of energy metabolism widely expressed in organs that need a large quantity of energy [47]. Recent studies have reported that intracellular cholesterol regulated the expression and activity of ERRα in breast cancer [28, 29]. However, whether the gefitinib and osimertinib resistance promoted by cholesterol depends on ERRα is unknown. Our work revealed that cholesterol increased and MβCD decreased ERRα expression. The effect of MβCD was counteracted by cholesterol supplement. Moreover, ERRα inhibition limited the effect of cholesterol by restoring cholesterol-induced gefitinib and osimeritinib resistance. Further investigation confirmed that ERRα inhibition increased the sensitivity of NSCLC cells to EGFR-TKIs through upregulating ROS level and arrested cell cycle in G0/G1 phase.

The mechanism by which cholesterol accumulation increases ERRα expression in gefitinib and osimertinib-resistant NSCLC cells is unknown. It has been reported that the activation of EGFR was linked to cholesterol level in lipid rafts [19]. The relationship between EGFR activation and ERRα expression has not been determined. Interestingly, EGFR reactivation and ERRα re-expression were observed synchronously in resistant cells under the treatment of gefitinib or osimertinib. The ERRα level was suppressed in NSCLC cells when p-EGFR expression was inhibited regardless of sensitivity to EGFR-TKIs. These data indicated the positive regulation of ERRα expression by EGFR signaling activation in the progress of TKIs resistance. Our study provided an evidence that accumulation of cholesterol recruited EGFR, Src assembly in lipid rafts and promoted interaction of the two proteins following by reactivation of EGFR/Src/Erk signaling pathway and re-expression of ERRα. Moreover, CHX chase assay showed gefitinib and osimertinib had no influence in the protein stability of ERRα. We speculated that the regulation might occur at the transcriptional level. UCSC Genome Browser Database and JASPAR database predicated SP1 as a transcription factor binding to the ERRα promoter region. SP1 is a zinc finger-type transcription factor which regulate various of genes in normal tissues and tumors [48]. Erk signaling has been reported as an upstream factor of SP1 [49]. In the current study, SP1 overexpression promoted ERRα expression and SP1 inhibition decreased ERRα expression in NSCLC cells. Blocking of EGFR/Src/Erk signaling suppressed nuclear translocation of SP1 and overexpression of SP1 blocked the inhibitory effect of EGFR/Src/Erk inhibitors on ERRα expression. In addition, elimination of cholesterol promoted gefitinib or osimertinib to restrain SP1 nuclear translocation. However, the effect was weakened by cholesterol supplement. Our observations illustrated that SP1 was a downstream factor of cholesterol/EGFR/Src/Erk axis and regulated ERRα expression. Moreover, SP1 was identified to promote ERRα transcription directly by binding to ERRα promoter region in NSCLC. Besides, our work suggested that pharmacological lowing cholesterol by lovastatin and inhibition of ERRα represented a viable strategy to overcome EGFR-TKIs in NSCLC.

## Conclusions

**Fig. 7:**
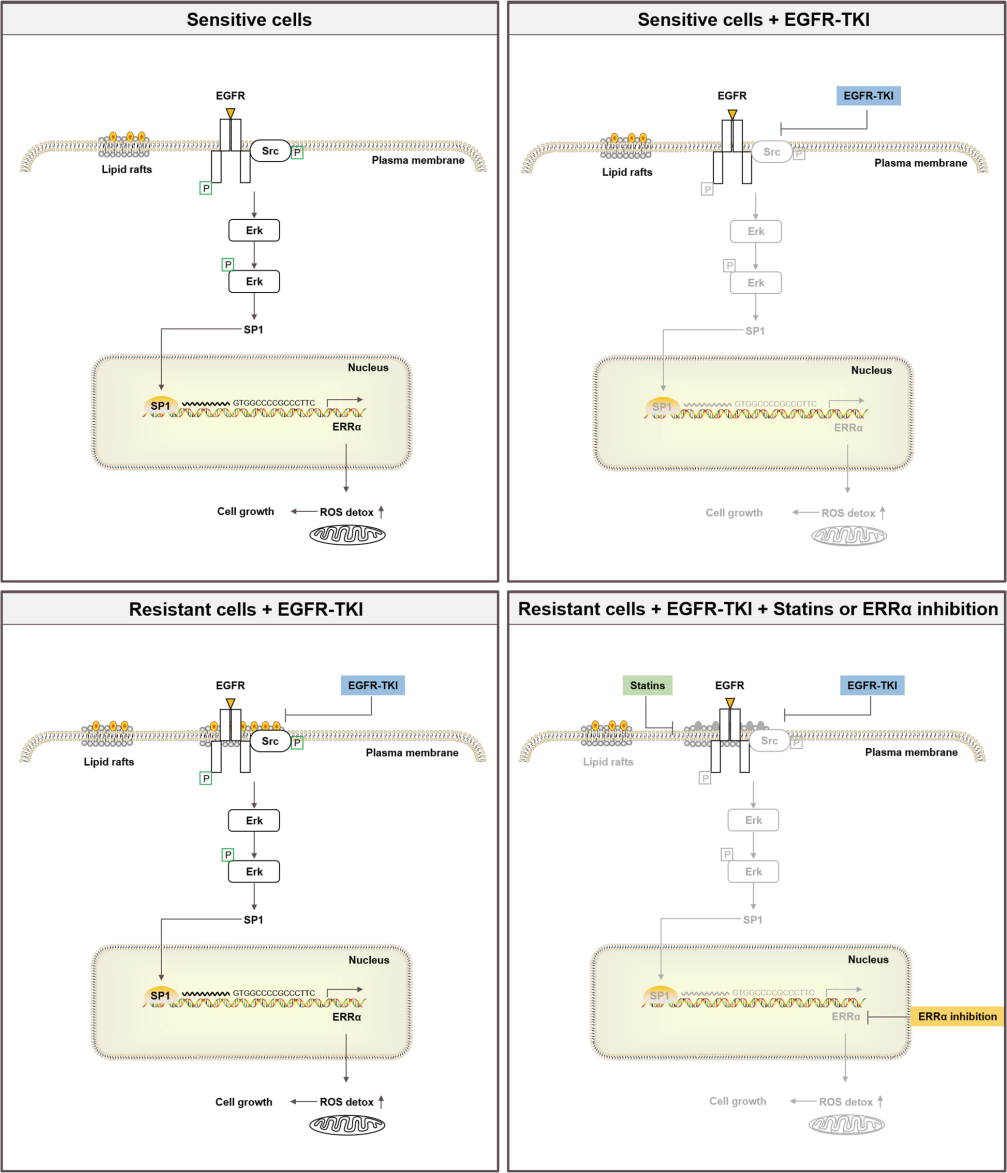
A working model of the role of cholesterol/EGFR/Src/Erk/SP1/ERRα axis in dictating the response of NSCLC cells to EGFR-TKIs and potential for cholesterol-lowing drugs or ERRα inverse agonist to counteract EGFR-TKIs resistance in NSCLC

In conclusion, the present study uncovers a common molecular mechanism sustaining resistance to both gefitinib and osimertinib that involves the re-expression of ERRα. Long exposure to gefitinib or osimertinib lead to cholesterol of lipid rafts accumulation in NSCLC thus provided a platform for EGFR and Src interaction and re-activation of EGFR/Src/Erk signaling pathway. SP1 nuclear translocation increased and promoted ERRα transcription by binding to its promoter. Re-expression of ERRα further elicits a ROS detox context enabling cell survival despite constant exposure to EGFR-TKIs. Importantly, this study demonstrates that pharmacological lowing of cholesterol and inhibition of ERRα restore gefitinib or osimertinib sensitivity in resistant cells and xenograft tumors in vivo (Fig. 7).

## Supplementary Information

**Additional file 1: Fig. S1.** ERRα is highly expressed in NSCLC and predicts poor prognosis of NSCLC patients. **a** The expression of ERRα in normal and lung adenocarcinoma patients. The data and *P* values were obtained from the OncoMine databas. **b** Kaplan–Meier analysis of ERRα (1487_at) expression in survival of Lung cancer patients. The data and *P* values were obtained from the Kaplan–Meier Plotter database (http://kmplot.com/analysis/index.php?p=background).

**Additional file 2: Fig. S2.** Cisplatin cannot regulate ERRα expression and EGFR-TKIs fail to influence ERRα protein stability. **a** ERRα expression was measured by Western blot in NSCLC cells cultured with cisplatin (HY-17394, MCE, New Jersey, USA) for 48 h. **b** Stability of ERRα protein was measured by CHX (SC0353, Beyotime Biotechnology, Shanghai, China) chase assay in PC-9 and H1975 cells cultured with gefitinib or osimertinib treatment. Then the ERRα protein half-life was analyzed.

**Additional file 3: Table S1.** Potential transcription factors were identified using the UCSC genome browser tracks in *homo sapiens* (hg38) for all profiles with a track score cutoff of 650 which equals *P* < 0.000001. **Table S2.** JASPAR database was used to predict possible transcription factor binding sites with Matrix ID, relative binding score and strand (with 90% cutoff). **Table S3.** The number of predicted transcription factor binding sites in the ERRα promoter region were counted.

## Data Availability

The datasets used during the current study are available from the corresponding author on reasonable request.

## Abbreviations

NSCLC: Non-small cell lung cancer
EGFR-TKIs: Epidermal growth factor receptor tyrosine kinase inhibitors
OS: Overall survival
PFS: Progression free survival
ERRα: Estrogen related receptor alpha
MβCD: Methyl-β-cyclodextrin
CHX: Cycloheximide
CHIP: Chromatin immunoprecipitation
IHC: Immunohistochemistry
RT-qPCR: Real-time quantitative polymerase chain reaction
CTB: Cholera Toxin Subunit B
