# Supplementary figures and images for: Cholesterol promotes EGFR-TKIs resistance in NSCLC by inducing EGFR/Src/Erk/SP1 signaling-mediated ERRα re-expression
Source: Mol Cancer. 2022 Mar 18;21:77. doi: 10.1186/s12943-022-01547-3 (PMC8932110; doi:10.1186/s12943-022-01547-3)

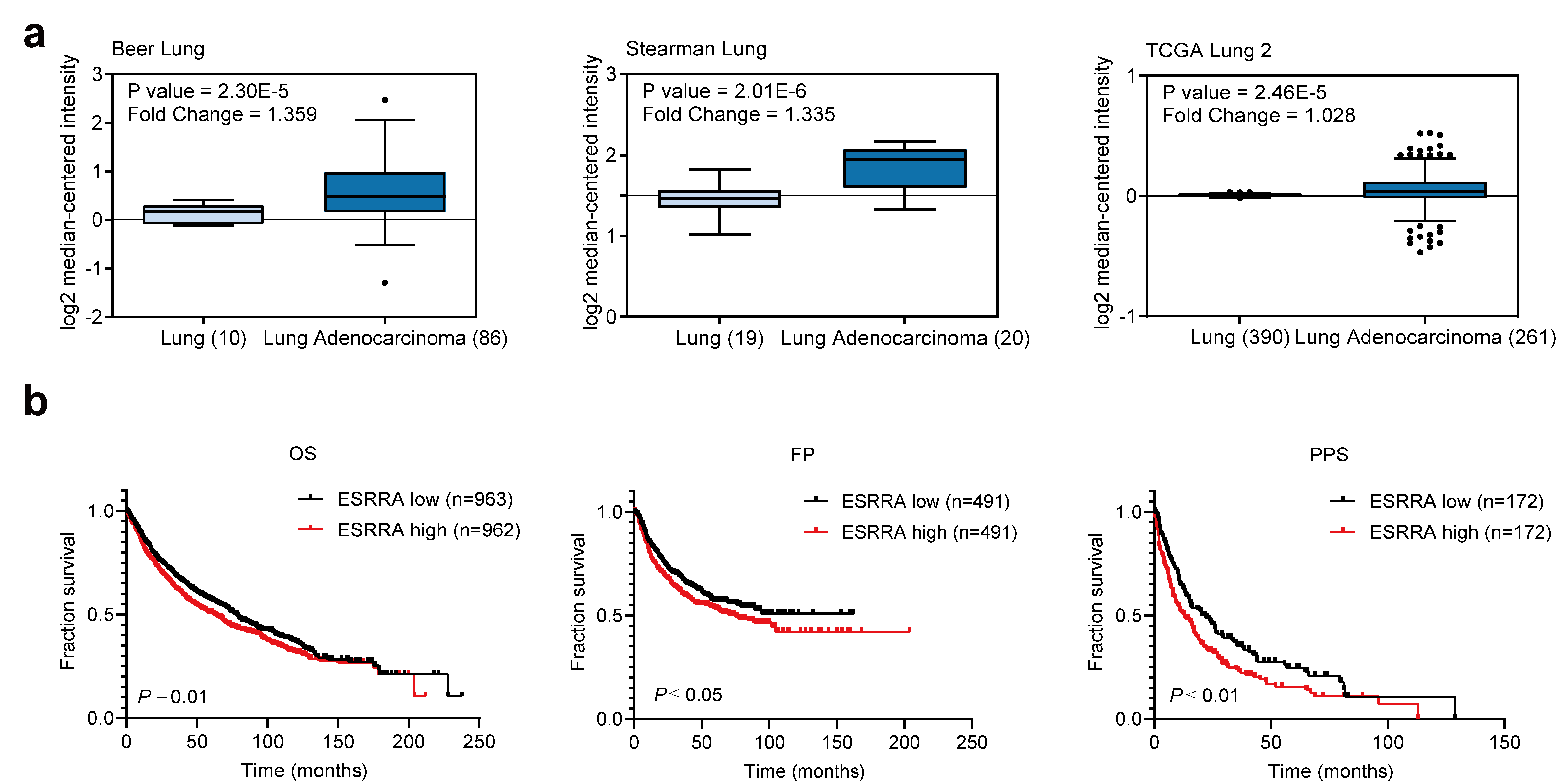

Supplement: Supplementary file 1 — Additional file 1: Fig. S1. ERRα is highly expressed in NSCLC and predicts poor prognosis of NSCLC patients. a The expression of ERRα in normal and lung adenocarcinoma patients. The data and P values were obtained from the OncoMine databas. b Kaplan–Meier analysis of ERRα (1487_at) expression in survival of Lung cancer patients. The data and P values were obtained from the Kaplan–Meier Plotter database (http://kmplot.com/analysis/index.php?p=background). [file 12943_2022_1547_MOESM1_ESM.tif]

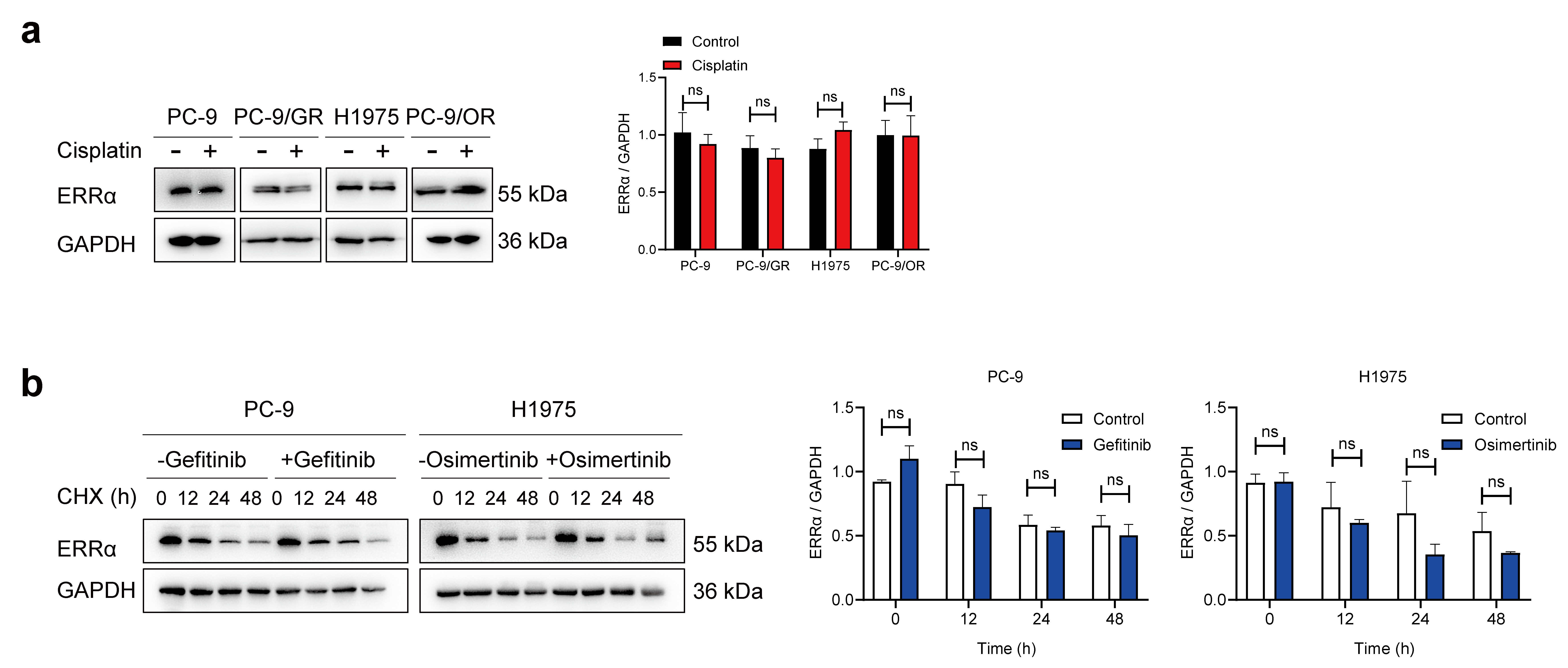

Supplement: Supplementary file 2 — Additional file 2: Fig. S2. Cisplatin cannot regulate ERRα expression and EGFR-TKIs fail to influence ERRα protein stability. a ERRα expression was measured by Western blot in NSCLC cells cultured with cisplatin (HY-17394, MCE, New Jersey, USA) for 48 h. b Stability of ERRα protein was measured by CHX (SC0353, Beyotime Biotechnology, Shanghai, China) chase assay in PC-9 and H1975 cells cultured with gefitinib or osimertinib treatment. Then the ERRα protein half-life was analyzed. [file 12943_2022_1547_MOESM2_ESM.tif]
