# Supplementary material for: Cholesterol promotes EGFR-TKIs resistance in NSCLC by inducing EGFR/Src/Erk/SP1 signaling-mediated ERRα re-expression
Source: Mol Cancer. 2022 Mar 18;21:77. doi: 10.1186/s12943-022-01547-3 (PMC8932110; doi:10.1186/s12943-022-01547-3)
Supplement: Supplementary file 3 — Additional file 3: Table S1. Potential transcription factors were identified using the UCSC genome browser tracks in homo sapiens (hg38) for all profiles with a track score cutoff of 650 which equals P < 0.000001. Table S2. JASPAR database was used to predict possible transcription factor binding sites with Matrix ID, relative binding score and strand (with 90% cutoff). Table S3. The number of predicted transcription factor binding sites in the ERRα promoter region were counted. [file 12943_2022_1547_MOESM3_ESM.docx]

**Supplementary Tables**

**Table S1.** The predicted transcription factors that regulate ERRα

| Item | Score | Position | Strand |
| --- | --- | --- | --- |
| CTCFL | 888 | chr11:64304476-64304489 | - |
| PLAG1 | 798 | chr11:64303901-64303914 | + |
| CTCF | 789 | chr11:64304475-64304493 | - |
| SP1 | 711 | chr11:64305478-64305488 | + |
| SP2 | 701 | chr11:64304816-64304830 | + |
| KLF16 | 676 | chr11:64305478-64305488 | + |
| NRF1 | 668 | chr11:64305430-64305440 | - |

Show only items with score at or above 650 which equals *P*< 0.000001

**Table S2.** JASPAR-predicted transcription factor binding sites in the ERRα promoter region

| Matrix ID | Name | Relative Binding Score | Strand |
| --- | --- | --- | --- |
| MA0079.3 | SP1 | 0.999999996 | + |
| MA1102.1 | CTCFL | 0.998108836 | - |
| MA0506.1 | NRF1 | 0.993741621 | - |
| MA0079.3 | SP1 | 0.986723525 | + |
| MA0741.1 | KLF16 | 0.978779943 | + |
| MA0079.3 | SP1 | 0.978747657 | + |
| MA0079.3 | SP1 | 0.977992111 | + |
| MA0079.3 | SP1 | 0.964299011 | + |
| MA0163.1 | PLAG1 | 0.963180893 | + |
| MA0079.3 | SP1 | 0.958484255 | + |
| MA0079.3 | SP1 | 0.956410443 | + |
| MA0741.1 | KLF16 | 0.951393384 | + |
| MA0079.3 | SP1 | 0.949228192 | - |
| MA0741.1 | KLF16 | 0.944612292 | + |
| MA0079.3 | SP1 | 0.944195618 | - |
| MA0506.1 | NRF1 | 0.944120494 | + |
| MA0079.3 | SP1 | 0.943346426 | + |
| MA0079.3 | SP1 | 0.941051568 | - |
| MA0139.1 | CTCF | 0.937839428 | - |
| MA0079.3 | SP1 | 0.936908946 | + |
| MA0741.1 | KLF16 | 0.930777223 | - |
| MA0079.3 | SP1 | 0.927091193 | + |
| MA0079.3 | SP1 | 0.92412245 | + |
| MA1102.1 | CTCFL | 0.91667649 | - |
| MA0741.1 | KLF16 | 0.914916999 | + |
| MA0079.3 | SP1 | 0.913409863 | + |
| MA0506.1 | NRF1 | 0.910921561 | + |
| MA0079.3 | SP1 | 0.909458806 | + |
| MA0516.2 | SP2 | 0.901228091 | + |

Show only items with relative binding score at or above 90%.

**Table S3.** The number of predicted transcription factor binding sites

| Matrix ID | Name | Number of transcription factor binding sites |
| --- | --- | --- |
| MA0079.3 | SP1 | 16 |
| MA0741.1 | KLF16 | 5 |
| MA0506.1 | NRF1 | 3 |
| MA1102.1 | CTCFL | 2 |
| MA0139.1 | CTCF | 1 |
| MA0163.1 | PLAG1 | 1 |
| MA0516.2 | SP2 | 1 |
